# Fatigue Effect on Low-Frequency Force Fluctuations and Muscular Oscillations during Rhythmic Isometric Contraction

**DOI:** 10.1371/journal.pone.0085578

**Published:** 2014-01-21

**Authors:** Yen-Ting Lin, Chia-Hua Kuo, Ing-Shiou Hwang

**Affiliations:** 1 Physical Education Office, Asia University, Taichung, Taiwan; 2 Department of Sports Sciences, University of Taipei, Taipei, Taiwan; 3 Department of Physical Therapy, College of Medicine, National Cheng Kung University, Tainan, Taiwan; 4 Institute of Allied Health Sciences, National Cheng Kung University, Tainan, Taiwan; University of California, Merced, United States of America

## Abstract

Continuous force output containing numerous intermittent force pulses is not completely smooth. By characterizing force fluctuation properties and force pulse metrics, this study investigated adaptive changes in trajectory control, both force-generating capacity and force fluctuations, as fatigue progresses. Sixteen healthy subjects (20–24 years old) completed rhythmic isometric gripping with the non-dominant hand to volitional failure. Before and immediately following the fatigue intervention, we measured the gripping force to couple a 0.5 Hz sinusoidal target in the range of 50–100% maximal voluntary contraction. Dynamic force output was off-line decomposed into 1) an ideal force trajectory spectrally identical to the target rate; and 2) a force pulse trace pertaining to force fluctuations and error-correction attempts. The amplitude of ideal force trajectory regarding to force-generating capacity was more suppressed than that of the force pulse trace with increasing fatigue, which also shifted the force pulse trace to lower frequency bands. Multi-scale entropy analysis revealed that the complexity of the force pulse trace at high time scales increased with fatigue, contrary to the decrease in complexity of the force pulse trace at low time scales. Statistical properties of individual force pulses in the spatial and temporal domains varied with muscular fatigue, concurrent with marked suppression of gamma muscular oscillations (40–60 Hz) in the post-fatigue test. In conclusion, this study first reveals that muscular fatigue impairs the amplitude modulation of force pattern generation more than it affects the amplitude responsiveness of fine-tuning a force trajectory. Besides, motor fatigue results disadvantageously in enhancement of motor noises, simplification of short-term force-tuning strategy, and slow responsiveness to force errors, pertaining to dimensional changes in force fluctuations, scaling properties of force pulse, and muscular oscillation.

## Introduction

When muscles are exhausted, they cannot generate enough force to achieve a target level because of a reduction in force-generating capacity. Muscular fatigue typically manifests as variations in the amplitude and spectrum of surface electromyograms (EMG); however, myoelectrical manifestations of a fatigued muscle vary with the load characteristics of a fatigue protocol [1], [2], such as static versus dynamic contractions [3] and maximal versus submaximal contractions [4], [5]. An invariant measure of change in an exhausted contraction appears to be increases in the size of force fluctuations [6], [7], traditionally viewed as enhancement of background noises in motor drive [8], [9]. Although a larger size of force fluctuations with fatigue progress may impair task quality, how time-dependent structure of force fluctuations varies with fatigue is little understood. The structure of force fluctuations can be indexed with different nonlinear analyses (such as entropy measures, detrended fluctuation analysis, recurrence quantification analysis, and so on), which describe the complexity of force fluctuations by assessing the degree of predictability of fluctuations over a force data stream [10], [11], [12]. For an un-fatigued muscle, the size and the complexity of force fluctuations are influenced by separate control processes [10], [11]. Increases in the complexity of force fluctuations do not necessarily undermine task quality, but they do reflect the engagement of error corrections. Force tracking with visual feedback results in better tracking congruency, smaller force fluctuations, and greater complexity of force fluctuations than force tracking without visual feedback [13], [14]. Also, in aged adults, force fluctuations are greater in size but less complex for constant-force level tasks [15].

Force fluctuations are ascribed to numerous discrete blocks of pulse elements in a force profile [11], [16]. The genesis of force pulse elements in a continuous force profile is related to the sampled processes, with which the central nervous system could intermittently approximate the ideal movement against feedback delays in the visuomotor system [17], [18]. Several studies have also emphasized the roles of the pulse elements in an additive accuracy control mechanism to remedy trajectory deviations with the feedback or feedforward process [16], [19], [20]. Force pulse elements are scaled and superimposed onto ideal force trajectory to tune force deviations from a priori standard [21]. A smoother force output with less fluctuation indicates a lower number of error corrections and successful blending of pulse elements [22]. We argue that the scaling of force pulses co-varies with adaptive changes in force fluctuations with fatigue development, underlying variations in peripherally derived afferent information [23], [24], [25], lowering of the recruitment threshold of motor units [26], [27], and variable firing rates of motoneurons [28], and so on.

The present study investigated fatigue effect on force-generating capacity and force-tuning strategy during rhythmic force-tracking, by characterizing organizational properties of the rhythmic force component of the target rate (ideal force trajectory) and force fluctuations (corrective actions). On account of target constraints to produce the criterion force, rhythmic force-tracking has higher task demand on force-tuning properties than a constant-load contraction [15], [29]. Rhythmic force-tracking with specific rate coding for the patterned muscle contraction [30], [31] exhibits more frequent and evident force adjustments than static contraction [29]. To our knowledge, research has not been conducted to investigate force-tuning strategies of a muscle following rhythmic fatiguing contraction with the size and the complexity of force fluctuations. Moreover, if responsiveness of force tuning is not fully a corollary of force-generating capacity, motor fatigue could differently impact accuracy and efficiency of force production. Another goal of this study was to link feature changes in force fluctuations with EMG oscillations in a fatigued muscle, as previous studies have shown that beta (13–20 Hz) and gamma (40–60 Hz) cortico-muscular rhythms are crucial for force stabilization during static and rhythmic isometric contraction [32], [33], [34]. Our main hypotheses were that 1) the fatigue effect would result in differential adaptations in rhythmic force component of the target rate (ideal force trajectory) and force fluctuations (corrective actions); 2) the size, the complexity, and spectral components of the force fluctuations would reduce after fatiguing contraction, and 3) fatigue-related modulation of force fluctuations would concur with suppression of muscular oscillations. The present findings could lend novel insight into the internal coding of motor commands for an exhausted muscle to tune force trajectory during rhythmic isometric contraction.

## Methods

Subjects. Sixteen subjects (20–24 years, all males) were convenience samples recruited from the department of the university. The subjects had completed a previous study in our laboratory, so that they were skilled at the rhythmic isometric gripping prior to the experiment. All subjects were self-reported as being right-handed, and none had symptoms or signs of neuromuscular diseases. The research project was approved by an authorized institutional human research review board of the Chung-Shan University Hospital, Taiwan. All subjects signed informed consents before the experiment in accordance with the Declaration of Helsinki.

**Figure 1:**
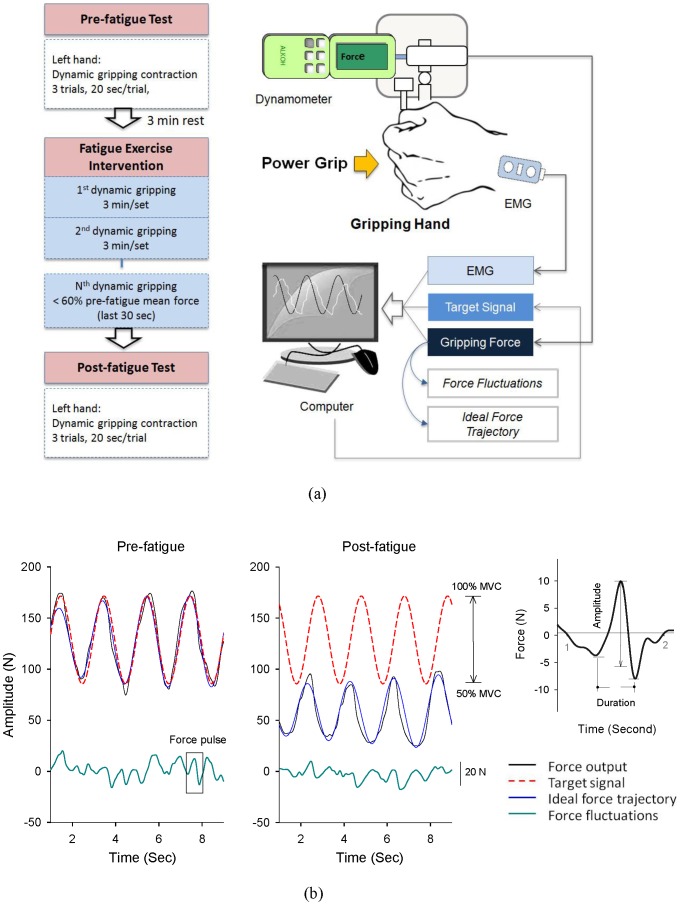
Experiment procedure, system setup, and data recording. (a) Schematic diagrams of the apparatus and fatigue protocol, (b) Typical recordings of force and target signals in the pre-fatigue and post-fatigue tests. The force signal was decomposed into a ideal force trajectory and a force pulse trace, visibly smaller after fatiguing contraction. Measures of amplitude and duration of a force pulse in the force pulse trace are displayed.

Experimental procedures. The subjects sat on a chair with their non-dominant (left) hands placed on the touch plate of a force gauge fixed firmly on the test table. The standard position for all subjects was shoulder flexion of 0 degrees, elbow flexion of 90 degrees, and the wrist in the neutral position. The subjects were informed that they would be using the non-dominant hand to complete three 20-second trials of isometric power gripping in a rhythmic manner in the pre-fatigue and post-fatigue tests, conducted prior to and following the fatigue intervention (Fig. 1(a)). The fatigue intervention was intended to cause a remarkable difference in mean force level of rhythmic force output between the pre-fatigue and post-fatigue tests. In the beginning of the experiment, the maximal voluntary contraction of power gripping was predetermined for the non-dominant hand with visual feedback, by selecting the peak force from three sets of 3-s maximal gripping contractions separated by 3 minutes each.

Following 3 minutes of rest, three trials of the pre-fatigue test were measured with inter-trial intervals of at least 1 minute. After that, the subjects performed the fatiguing protocol, which contained several contraction trials of 3-minute rhythmic isometric power gripping similar to that used in the pre-fatigue test. There was no resting period between the trials in the fatiguing protocol. The fatiguing protocol continued until the mean force output in the last 30 seconds of the last trial was lower than 60% of the mean force level of the pre-fatigue test. Three successive trials of the post-fatigue test, without resting periods, were completed similarly to the pre-fatigue test.

The force-tracking paradigm used in the pre-fatigue test, fatigue exercise intervention, and the post-fatigue test was rhythmic isometric power gripping. The subjects manipulated the position of a manual cursor on a computer monitor, which was linearly proportional to the force exerted by the gripping hand. The subjects performed rhythmic isometric contraction in the range of 50%–100% of maximal gripping force in order to couple the manual cursor with a 0.5 Hz sinusoidal target wave. The target signal moved vertically in a range of 7.2° of visual angle (i.e., 3.6° above and 3.6° below the eye level on the screen). The manual cursor and the target curve were displayed on the computer monitor as on-line visual guidance for force tracking. Prior to the experiment, the subjects were allowed for a few practice trials to get familiar with rhythmic gripping following by a rest period without causing fatigue. In case of motor fatigue, the subjects could not exert sufficient force output to keep with the target trace, especially when the mean force level and range of force modulation were smaller than those of the pre-fatigue condition.

A digital force gauge (sensitivity: 0.01 N, bandwidth = DC-1 kHz, Model: 9820P, AIKOH, Japan) connected to an analog amplifier (Model: PS-30A-1, Entran, UK) was used to measure the gripping force that the subjects exerted on the touch plate. The bipolar surface electrode units (1.1 cm in diameter, gain = 365, CMRR = 102 dB, Imoed Inc., USA) were used to measure the left flexor digitorum superficialis (FDS). The muscle activity of the FDS was recorded by placing the electrode at an oblique angle approximately 4 cm above the wrist on the palpable muscle mass. The target signal, force output, and EMG were sampled at 1 kHz using a custom program on a Labview platform (National Instruments, Austin, TX, USA).

Data processing. Gripping force was down-sampled to 100 Hz in off-line analysis and then conditioned with a low-pass filter (cut-off frequency: 6 Hz) [20], such that physiological tremor in 8–12 Hz was excluded. The exclusion of physiological tremor in 8–12 Hz was because it is an involuntary movement irreverent to corrective actions. Only force components of lower frequency below 4 Hz could be considered as sensorimotor process for voluntary movement control [15], [20], [21]. The mean gripping force in an experimental trial was thus obtained by averaging the conditioned gripping force profile in the pre-fatigue and post-fatigue tests. The tracking congruency was assessed with the correlation coefficient between the target trajectory and gripping force profile. Then the force profile was further decomposed into two time series, the ideal force trajectory and the force pulse trace (Fig. 1(b)) [20], [35]. The ideal force trajectory was a smooth 0.5 Hz sinusoidal wave that approximated the force profile of rhythmic isometric gripping in amplitude. Therefore, the ideal force trajectory represented an ideal force trajectory with the identical spectral component to target rate. In contrast, the force pulse trace was an irregular component of gripping force and a major source of force fluctuations. Force pulse trace could be obtained by conditioning the force output with a zero-phasing notch filter that passes all frequencies except for a target rate at 0.5 Hz. The transfer function of the notch filter (*H(z)*) in this study was 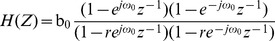, *r* = .9975, *ω*_0_ = *π*/360. We obtained the ideal force trajectory by subtracting the gripping force from the force pulse trace (RMS__FP_). The amplitudes of the ideal force trajectory profile (RMS__IF_) and force pulse trace were calculated by root mean square. The amplitude ratio (R_IF/FP_) of the ideal force trajectory to force pulse trace was the RMS value of the ideal force trajectory divided by the RMS of the force pulse trace. R_IF/FP_ estimated the ratio of the regular component of gripping force relative to the irregular component of gripping force.

The spectral distribution of the force pulse trace was calculated with the Welch method. A Hanning window was applied to each artifact-free 2.048-second epoch of the force pulse trace with an overlap of 0.512 seconds. The spectral density was calculated using a fast Fourier transform, and the spectral resolution was 0.02 Hz. Mean frequency and mode frequency were determined from the spectral profile of the force pulse trace. Spectral dispersion of the force pulse trace was defined as the spectral ranges between the 10th and 90th percentiles of the power spectra. We also quantified the complexity variations in a force pulse trace with multi-scale entropy (MSE), or sample entropy (SampEn) across different time scales [36], [37]. The calculation of the MSE consisted of three steps. First, obtain the coarse-grained sequences of down-sampled force pulse trace 
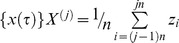
, where {z_1_, z_2_, …, z_N_} is the time series of force pulse trace and τ is the time scale. Next, sample entropy {*SampEn(m,r,N)* ^(τ)^, _τ = 1,2,…40_} for each coarse-grained sequence {*X(τ)*} were calculated. Sample entropy measures the negative natural logarithm of an estimate of the conditional probability that epochs of length m that match point-wise within a tolerance level (*r*) also match at the next point. The mathematical formula of sample entropy was 
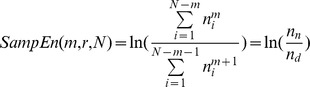
, where *r* = 20% of where the standard deviations of *X(τ)* and *m* = 2. Finally, the MSE areas in low and high time scales were defined as summation of the SampEn in the time scales 1–5 and 25–40, respectively. That is, 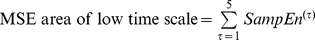 and 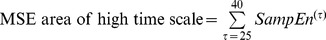. MSE area has been shown to be robust in measuring the complexity of biological time-series [36], [37], with a higher MSE area indicating a noisier structure. The time scale for MSE was 10 ms after down-sampling (100 Hz).

The spatial and temporal parameters of individual force pulses were characterized. Pulse amplitude was defined as the difference between a local maximum and the average value of the two nearest minima (Fig. 1(b)) [20], [35]. To exclude insignificant force pulses contributed by random noise, this study defined the threshold pulse amplitude as the upper limit of a 95% confidence interval of the pulse amplitude in the relaxed condition before the fatiguing contraction. Only force pulses exceeding the threshold amplitude were considered meaningful. The pulse duration of these meaningful force pulses was the time between two successive local minima in the force pulse trace. Mean kurtosis and mean skewness of the pulse amplitude and pulse duration of the tracking trials in the pre-fatigue and post-fatigue tests were averaged.

Band-pass filters were used to condition the EMG of the left FDS muscles (pass band: 1∼400 Hz). The RMS values and mean frequencies of the EMG for the FDS muscles were determined in the pre-fatigue and post-fatigue tests. In addition, we estimated the power spectrum of the rectified EMG, as rectification of surface EMG is thought to highlight the spectral peaks that symbolize common oscillatory inputs or the mean firing rate of an active muscle [38]–[40]. The spectral profiles of rectified EMG of the three trials in the pre-fatigue and post-fatigue tests were averaged and normalized with the mean spectral amplitude to reduce population variability. Spectral peaks in the alpha (8–12 Hz) and gamma (40–60 Hz) bands were obtained from the mean standardized spectral profile of rectified EMG. The power spectra of the un-rectified and rectified EMG were estimated with the Welch method and a fast Fourier transform (Hanning window: 2.048-second epoch with an overlap of 0.512 seconds). The spectral resolution for FDS EMG was 0.244 Hz. Signal processing was completed using a MATLAB script (The Mathworks Inc., Natick, MA, USA).

Statistical analyses. The force variables (ideal force trajectory and force pulse trace) as well as EMG variables of the three trials in the pre-fatigue and post-fatigue tests were averaged for each subject. Hotelling's T^2^ statistics and post-hoc analysis with Bonferoni corrections were used to examine the significance of parametric changes due to the fatigue effect, by contrasting tracking outcomes (mean gripping force and tracking congruency), force amplitude variables (RMS__IF_, RMS__FP_, and R_IF/FP_), EMG variables (RMS, mean frequency, alpha spectral peak, and gamma spectral peak), spectral features of force pulse trace (mean frequency, mode frequency, and spectral dispersion), complexity of force pulse trace (MSE areas in high and low time scales), temporal force pulse characteristics (pulse duration, duration kurtosis, and duration skewness), and spatial force pulse characteristics (pulse amplitude, amplitude kurtosis, and amplitude skewness) between the pre-fatigue and post-fatigue tests. Pearson's correlation was used to examine the significance of correlation between force pulse metrics and the size (RMS of force pulse trace)/the complexity (MSE areas) of force variability in the pre-fatigue and post-fatigue conditions. Statistical analysis was completed with the SPSS 15.0 statistical package (SPSS Inc., Armonk, NY, USA). The significance level was set at 0.05. Data reported in the texts, figures, and tables are presented as mean ± standard error (SE).

## Results

Hotelling's T^2^ statistics suggested a fatigue effect on force variables (Wilks's Λ = .075, *P*<.001). The mean force level was smaller in the post-fatigue test (71.15±3.68 N) than in the pre-fatigue test (119.65±5.04 N)(*P*<.001). Namely, mean force level in the post-fatigue test was 59.79±2.16% of that in the pre-fatigue test. Tracking congruency in the post-fatigue test (.266±.054) was markedly smaller than that in the pre-fatigue test (.757±.028)(*P*<.001). Force measures confirmed failure of the gripping task in the post-fatigue test.

Figure 1(b) contrasts the force profile, ideal force trajectory, and force pulse trace between the pre-fatigue and post-fatigue tests from a typical subject. This plot clearly reveals that the capacity of rhythmic force regulation and mean force level in the post-fatigue test were lower than those in the pre-fatigue test. Table 1 presents the means and standard errors for the amplitudes of ideal force trajectory and force pulse trace, as well as the amplitude ratio of ideal force trajectory to force pulse trace (R_IF/FP_) before and after the fatiguing exercise. Hotelling's T^2^ statistic suggested a significant fatigue effect on those force pulse variables (Wilks'Λ = .29, *P* = .001). RMS values of the ideal force trajectory and force pulse trace were significantly smaller in the post-fatigue test (*P*<.001). It is worth noting that R_IF/FP_ was smaller in the post-fatigue test (pre-fatigue: 5.36±0.18 vs. post-fatigue: 4.35±0.22)(*P*<.01), specifying a non-parallel amplitude reduction for the ideal force trajectory and force pulse trace due to fatigue, with greater suppression on the ideal force trajectory than on the force pulse trace.

**Table 1:** The contrast of amplitude variables of the ideal force trajectory and force pulse between pre-fatigue and post-fatigue.

Amplitude variable^1^	Pre-fatigue	Post-fatigue
RMS__IF_ (N)^3^	29.57±2.04	15.30±1.89^***^
RMS__FP_ (N)^4^	5.59±0.40	3.34±0.30^***^
R_IF/FP_ 5	5.36±0.18	4.35±0.22^++^
*Statistics*	Λ = 0.290, *P* = .001^2^	

1 Values were presented as mean ± se.

2 Post-hoc for pre-fatigue vs. post-fatigue (^***^: post-fatigue<pre-fatigue, *P*<.001; ^++^: post-fatigue<pre-fatigue, P<.01).

3 RMS__IF_: root mean square of ideal force trajectory.

4 RMS__FP_: root mean square of force pulse trace.

5 R_IF_/_FP_ denotes amplitude ratio of the ideal force trajectory to force pulse trace.

**Figure 2:**
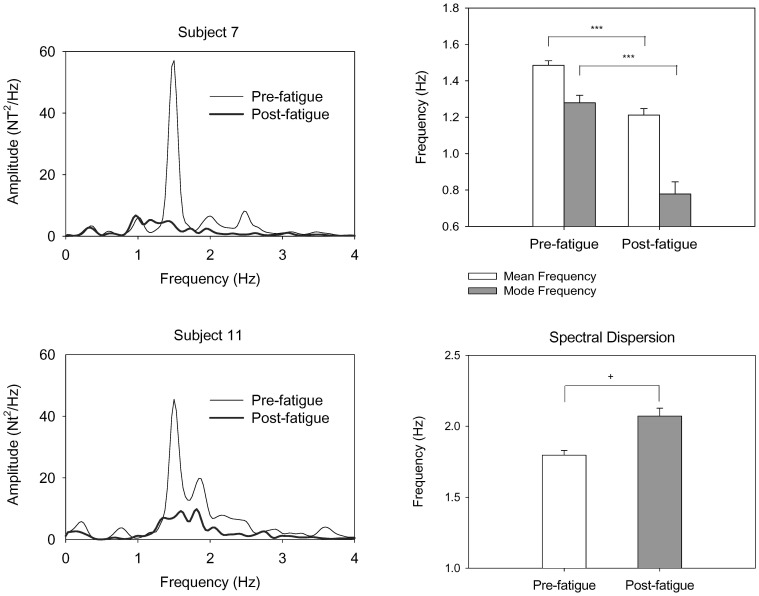
Contrasts of spectral features of force pulse trace between the pre-fatigue and post-fatigue tests. Spectral profiles of force pulse trace for two typical subjects are shown in the left plots. Fatigue effect on mean frequency, mode frequency and spectral dispersion of force pulse trace is summarized in the right plots. (^***^: Post-fatigue<Pre-fatigue, *P*<.001; ^†^: Post-fatigue>Pre-fatigue, *P*<.05).

**Figure 3:**
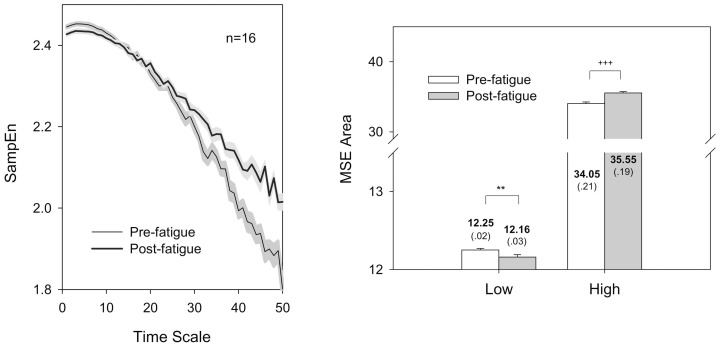
Contrasts of SampEn versus time scales and MSE area between pre-fatigue and post-fatigue tests. Time scale for MSE plot in temporal domain is 10(^**^: Post-fatigue<Pre-fatigue, *P*<.01; ^†††^: Post-fatigue>Pre-fatigue, *P*<.001)(SampEn: sample entropy; MSE: multi-scale entropy).

The left plots in Figure 2 show spectral variations in the force pulse trace from two typical subjects after the fatigue exercise intervention. Hotelling's T^2^ statistic showed that spectral features of the force pulse trace were subject to the fatigue effect (Wilks' Λ = 0.280, *P*<.001). The mean frequency and mode frequency of the force pulse trace in the post-fatigue test were much lower than those in the pre-fatigue test (mean frequency: pre-fatigue: 1.48±0.13 Hz, post-fatigue: 1.21±0.17 Hz)(mode frequency: pre-fatigue: 1.28±0.22 Hz; post-fatigue: 0.78±0.32 Hz)(*P*<.001)(Fig. 2, upper right). In addition, the force pulse trace in the post-fatigue test had a wider spectral dispersion than did that in the pre-fatigue test (*P* = .024)(Fig. 2, lower right). Figure 3 shows the results of multi-scale entropy (MSE) analysis to contrast the complexity of the force pulse traces before and after the fatigue intervention. The SampEn curves of the force pulse traces were visibly different for the pre-fatigue and post-fatigue tests (Fig. 3, left). The complexity of the force pulse traces in different time scales were estimated in terms of the area under the SampEn curves. The results of Hotelling T^2^ statistics suggested a significant fatigue effect on MSE areas of the low time scale (1–5) and the high time scale (25–40)(Wilks' Λ = 0.370, *P* = .001), though they were modulated in an opposite manner. The MSE area of the low time scale in the post-fatigue test was smaller (had greater regularity) than that of the pre-fatigue test (*P*<.01), whereas the MSE area of the high time scale in the post-fatigue test was larger (had greater complexity) than that in the pre-fatigue test (*P*<.001)(Fig. 3, right).

Scaling properties of individual force pulses before and after the fatigue intervention exercise are contrasted in Table 2. Spatial scaling of force pulses was subject to the fatigue intervention (Wilks' Λ = .183, *P*<.001), with a smaller pulse amplitude in the post-fatigue test (*P*<.001). Fatigue intervention did not significantly alter the amplitude kurtosis (*P* = .714) or amplitude skewness of force pulses (*P* = .208). Temporal scaling of force pulses was also subject to the fatigue intervention (Wilks' Λ = .279, *P* = .001). The pulse duration was smaller in the post-fatigue test than in the pre-fatigue test (*P*<.001). Moreover, fatigue intervention also led to significant enhancements of duration kurtosis (*P* = .001) and duration skewness (*P* = .003) in the post-fatigue test. Table 3 shows the results of Pearson's correlation between the size of a force pulse and force pulse metrics in the pre-fatigue and post-fatigue tests. In the pre-fatigue test, force pulse RMS was related to pulse amplitude (*r* = .935, *P*<.001). In addition to pulse amplitude (*r* = .958, *P*<.001), force pulse RMS was a function of pulse duration (*r* = .576, *P* = .019) in the post-fatigue test. Table 4 shows Pearson's correlation between the force pulse complexity and statistical properties of the force pulse variables. In the pre-fatigue condition, the MSE area of the force pulse was not related to the statistical properties of the force pulse variables, except for a significant correlation between duration skewness and MSE area in high time scales (*r* = .537, *P* = .032). In the post-fatigue test, however, MSE areas of force pulses in low and high time scales were negatively related to skewness and kurtosis of pulse amplitude (*r* = −.499∼−.699, *P*<.05).

**Table 2:** Hotelling's T^2^ statistics for force pulse variables in the pre-fatigue and post-fatigue tests.

Force pulse variable^1^	Pre-fatigue	Post-fatigue
Amplitude (N)	9.48±0.56	4.60±0.45^***^
Amplitude Kurtosis	2.99±0.09	3.09±0.14
Amplitude Skewness	0.56±0.05	0.63±0.05
Duration (Sec)	0.41±0.01	0.36±0.01^***^
Duration Kurtosis	0.46±0.07	3.13±0.10^++^
Duration Skewness	0.46±0.04	0.73±0.04^++^
*Statistics*	Λ_amplitude_ = 0.183, *P* = .000^2^	
	Λ_duration_ = 0.279, *P* = .001^2^	

1 Values were presented as mean ± se.

2 Post-hoc for pre-fatigue vs. post-fatigue (^***^: post-fatigue<pre-fatigue, *P*<.001; ^++^: post-fatigue>pre-fatigue, P<.01).

**Table 3:** Pearson's correlation coefficients between the size of force fluctuations and force pulse metrics.

(n = 16)	Pre-fatigue RMS__FP_ 1	Post-fatigue RMS__FP_ 1
Amplitude	r = .935, *P* = .000***	r = .958, *P* = .000***
Duration	r = .211, *P* = .432	r = .576, *P* = .019*

1 RMS__FP_ represents root mean square of force pulse trace.

* : *P*<.05;

*** : *P*<.001.

**Table 4:** Pearson's correlation coefficients between the complexity of force fluctuations and statistical properties of force pulse.

	Pre-fatigue		Post-fatigue	
(n = 16)	MSE__LTS_ 1	MSE__HTS_ 2	MSE__LTS_ 1	MSE__HTS_ 2
Amplitude Kurtosis	r = −.192, *P* = .477	r = .011, *P* = .969	r = −.634, *P* = .008**	r = −.499, *P* = .049*
Amplitude Skewness	r = −.462, *P* = .072	r = .355, *P* = .177	r = −.699, *P* = .003**	r = −.518, *P* = .040*
Duration Kurtosis	r = −.225, *P* = .402	r = .078, *P* = .774	r = .099, *P* = .716	r = .106, *P* = .695
Duration Skewness	r = −.066, *P* = .808	r = .537, *P* = .032	r = −.034, *P* = .901	r = .010, *P* = .970

1 MSE__LTS_ represents multi-scale entropy area of low time scale 1–5.

2 MSE__HTS_ represents multi-scale entropy area of low time scale 25–40.

* : *P*<.05;

** : *P*<.01.

**Figure 4:**
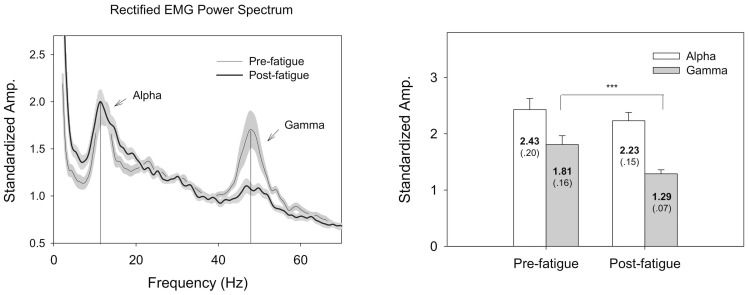
Fatigue effect on pooled spectral profiles and spectral features of the rectified EMG. Two spectral peaks in the 8–12 Hz and 40–60 Hz bands were noted, and the 40–60 Hz spectral peak was visibly suppressed after fatiguing exercise. (^***^: Post-fatigue<Pre-fatigue, *P*<.001).

After the fatigue exercise intervention, the results of Hotelling T^2^ statistics suggested a significant fatigue effect on EMG variables (Wilks's Λ = .274, *P* = .002). Post-hoc analysis suggested that the root mean square of EMG (pre-fatigue: 44.79±5.23 µV; post-fatigue: 14.93±2.4 µV) and mean frequency of un-rectified EMG (pre-fatigue: 56.51±1.91 Hz; post-fatigue: 48.61±2.15 Hz) decreased after the fatigue intervention (*P*<.001). Reductions in the mean frequency and amplitude of FDS EMG suggested a fatigued state of the muscle in the post-fatigue test. Figure 4 contrasts the pooled power spectrum of the rectified EMG and mean spectral peaks of the FDS muscle between the pre-fatigue and post-fatigue tests. Two prominent spectral peaks in the alpha (8–12 Hz) and gamma bands (40–60 Hz) were enhanced with the rectification process (Fig. 4, left). Post-hoc analysis revealed that the standardized amplitude of gamma muscular oscillation in the post-fatigue test (1.29±0.07) was significantly smaller than that in the pre-fatigue test (1.81±0.16)(*P* = 0.01)(Fig. 4, right). However, the alpha muscular oscillation was not significantly affected by the fatigue intervention (*P* = 0.555).

## Discussion

The designated fatiguing protocol led to task failure, as evidenced with the EMG (reductions in amplitude and mean frequency) and force behavior (declines in mean force level and tracking congruency) variables. We presented several novel findings on fatigue-related modulations of force fluctuations and muscular oscillation. Muscular fatigue impaired the balance between regular and irregular force components (R_IF/FP_), as a result of more pronounced amplitude suppression on the ideal force trajectory than on the force pulse trace. Muscular fatigue altered force-tuning strategies during rhythmic isometric contraction, underlying subsidence in higher spectral components and changes in complexity in force fluctuations. Fatigue also modified statistical properties of force pulse metrics, such that the size and the complexity of force fluctuations were differently represented by scaling of force pulses in the pre-fatigue and post-fatigue tests. The changes in force fluctuation properties co-varied with suppression of gamma muscular oscillation by the fatigue effect.

### Fatigue-related variations in ideal force trajectory and force pulse trace

In this study, the force outputs during rhythmic isometric gripping were dichotomized into the ideal force trajectory and force pulse trace. One of the methodological merits of the dichotomy is to firstly specify imbalance between ideal and irregular force components due to motor fatigue. Akin to kinematic submovements [17], [20], [35], force pulse trace of irregular nature represents many corrective attempts to remedy tracking mismatches, whereas ideal force trajectory symbolizes a priori force drive to couple rhythmic target movement. Also, ideal force trajectory is reminiscent of force command stabilizing the referent configuration trajectory, and force pulse trace is the emergent pattern complementary to the target goal, according to the lambda-hypothesis [41], [42]. Central to the interpretations, we noted reduction in the capacities to produce force in a rhythmic manner and to tune force trajectory for suppression of the ideal force trajectory and force pulse trace in the post-fatigue test (Table 1). A smaller R_IF/FP_ in the post-fatigue test further validated a more pronounced amplitude suppression on the ideal force trajectory due to motor fatigue (pre-fatigue: 5.36±0.18 vs. post-fatigue: 4.35±0.22). The non-parallel inhibition provides novel evidence that force fine-tuning is not a completely corollary of rhythmic force-generating capacity. Ideal pattern generation and error correction of force production were likely to be separated control processes, such that ideal force trajectory and force fluctuations were differentially affected by motor fatigue. According to the optimal feedback control framework [43], ideal force trajectory resembles to an efference copy of the force command (or open-loop control) used to state estimates of the force-tracking maneuver and force fluctuations reflect the solution to reproduce target kinematic pattern in an optimization process [43], [44]. In view of amplitude suppression, the ability to fine-tune force trajectory was less damaged by the fatiguing protocol than the capacity to generate rhythmic force was. Previous studies using static fatigue protocol have shown that fatigue increases in relative force variability [45], [46]. Our finding generalized the concept to rhythmic isometric contraction in terms of reduced R_IF/FP_ in the post-fatigue condition, implying the probability to miss the target increases if the same force pattern is planned during fatigue.

Changes in force pulse trace in the complexity and spectral features also lend novel insight into force trajectory control in a fatigued muscle. In the pre-fatigue test, a force pulse trace was composed of stereotypical force pulses with the major power spectrum spanning below 2 Hz (Fig. 2, left) [15], [16]. In case of motor fatigue, the force pulse trace was downward modulated to a lower spectral band below 1 Hz (Fig. 2, right). It is known that central- or muscular-based perception could be impaired by motor fatigue [47], [48].The slower oscillations in the force pulse trace, a biomarker of inability to produce sufficiently frequent corrective responses, was partly attributable to lacking timely feedback of real motor status. Besides, motor fatigue brought about marked complexity changes in force fluctuations (Fig. 3). In a fatigued state, the MSE area at low time scales of 1–5 became smaller, reminiscent of an increase in short-range correlation commonly observed in changed physiological complexity with aging and diseases [49]. An inherent part of transient loss of complexity in the force pulse trace is speculated to be an increase in the number of motoneurons d more than once in a short interval (doublets or triplets) during fatiguing contractions [50], a compensatory mechanism to escalate the central excitatory outflow to offset fatigue-induced increases in peripheral conduction impedance [51], [52]. According to the MSE algorithm, brief force events caused by short-term repetitive discharges of motoneurons could be counted as matching each other, when force pulse data were less coarse-grained at small time scales. The reduction in force fluctuation irregularity at low time scales functionally indicates a short-term simplification of trajectory control. Running counter to that at the low time scales, the enhancement of the MSE area at high time scales (25–40) in the post-fatigue test suggested that the long-range correlation of the force pulse trace were reduced. In terms of MSE area, the complexity increment of long-range force fluctuations seems to overpower the decrease in short-range force fluctuation (Fig. 3, left), comparable with an increase in force irregularity (increase in ApEn) for older adults during a sinusoidal force task [15]. Fatigue-related increase in long-range complexity could result from motor noises following fatigue [11], and additive randomness in force pulse data was mathematically invariant to more coarse-grained averaging using large time scales [36], [37]. MSE analysis reveals an opposite trend of force fluctuation complexity in low and high time scales. The fact speaks for interaction of at least two regulatory systems, which operate over a wide range of temporal scales underlying force tuning of a fatigued muscle.

### Temporal and spatial scaling of force pulse during fatigued rhythmic isometric gripping

The scaling properties of individual force pulses were also subject to the fatigue effect. In addition to an apparent inhibitory modulation on force pulse (fundamental elements of force output), we also note different representations of force fluctuations with force pulse metrics due to fatigue effect. In particular, the size of force fluctuations in the post-fatigue test became a function of pulse duration that was originally independent of force fluctuations for an un-fatigued muscle (Table 3). On account of the use of intermittent visual feedback for a visuomotor task [16], [18], [19], the fact that temporal scaling became a function of the size of force fluctuations seemingly related to increasing visual mismatches between the force output and target signal following fatigue-induced strength decrease (Fig. 1(b)). The feedback blur could add to extra computational load to remedy tracking deviations [44], such that the size of force fluctuations depended in part on temporal scaling of force pulses during the exhausted force-tracking. Fatigue also altered representation of the complexity of force fluctuations with force pulses (Table 4). The complexity of force pulse traces at high time scales was positively related to the temporal scaling property of force pulses (duration skewness) in the pre-fatigue test; however, the complexity of force pulse traces was negatively related to spatial scaling properties of force pulses (amplitude kurtosis and amplitude skewness) in the post-fatigue test. Despite that no known physiological mechanisms can directly explain such a variation in scaling properties due to fatigue, our findings clearly revealed that the size and complexity of force fluctuations were more constrained by known statistical properties of force pulse metrics after fatigue.

### Fatigue effect on oscillatory muscular activities during rhythmic isometric gripping

In addition to force fluctuation properties, waning of gamma EEG oscillation (40–60 Hz) was noted as a new EMG manifestation of motor fatigue during rhythmic isometric contraction (Fig. 4). Research has shown that corticomuscular coherence in the gamma band manifested with phasic movement [32], [54] and repetitive isotonic contraction [34]. For an un-fatigued muscle, gamma synchrony is believed to involve in global alertness to integrate sensory-motor information when a motor task entails temporal control of phasic patterns [32], [33], [54]. Although we did not directly measure the corticomuscular coherence (rectified EMG-EEG coherence), it was likely that spectral peaks of the rectified EMG could be muscular oscillations in the peripheral part of corticomuscular coherence [32], [53]. Consequently, it was not surprising to observe a prominent gamma muscular oscillation in the pre-fatigue test for successful execution of rhythmic isometric gripping. In post-fatigue test, the waning of gamma muscular oscillation indicates a failure of rapid recalibration of the neuromuscular plant demanded by the rhythmic force output, including reduced capacities in force pattern generation and tracking mismatches for rhythmic contraction. Another prominent muscular oscillation during rhythmic isometric gripping was in the alpha band (8–12 Hz), which could be physiological/force tremor [1], [2] or coherent modulation of motor unit discharge during quasi-sinusoidal isometric contraction [55]. However, alpha muscular oscillation is not susceptible to the fatigue effect, when EMG amplitude was normalized.

### Some methodological considerations

First, contrary to a focused definition of muscle fatigue by quantifying the decline in maximal force capacity [56], [57], the present study used a non-classical fatigue measurement to characterize and force generation pattern and force fluctuations at the moment of task failure. Hence, more exactly, changes in force behavior identified in this study reflect physiological mechanisms associated with inability of continuing force-tracking maneuver [57]. Next, because the subjects failed to keep in line with the target signal in the post-fatigue trials, fatigue-related changes in force behavior might be affected by feedback blur that added to extra computational load on the control of force accuracy during exhausted tracking [44]. Ideally, to specify strength decrement, the target signal should be timely adjusted to force performance for feedback accuracy. In practice, it was technically difficult to display optimal target trace based on prediction of varying fatigue states across individuals. Finally, this study required to produce force oscillations up to 100% MVC, which might exhaust the subjects and biased force measures in the pre-fatigue trials. However, we noted that almost all the subjects could successfully achieve the target goal, because tentative maximal effort during rhythmic force-tracking was less arduous than MVC that was determined by averaging 3-second sustained maximal isometric contraction.

## Conclusions

The present study adds to the increasing body of literature on force control in a fatigued muscle by characterizing force fluctuation properties and muscular oscillation. We firstly revealed that motor fatigue led to more amplitude suppression on ideal force pattern than on force fluctuations. Motor fatigue also results in structural changes in force fluctuations due to rescaling of force pulses in the temporal and spatial domains. A spectral shift of the force fluctuations to lower frequency bands and complexity changes in force fluctuations suggest that a fatigued neuromuscular plant with enhanced motor noises inclines to use a simplified force-tuning strategy, so that the plant is not responsive to rapid trajectory deviations for rhythmic force outputs. Suppression of 40–60 Hz muscular oscillation is likely the source of the characteristic changes in force behaviors, underlying lack of a sensible motor drive to produce rhythmic force output.
